# Sensitivity to Rocuronium-Induced Neuromuscular Block and Reversibility with Sugammadex in a Patient with Myotonic Dystrophy

**DOI:** 10.1155/2012/107952

**Published:** 2012-04-09

**Authors:** Akihiro Kashiwai, Takahiro Suzuki, Setsuro Ogawa

**Affiliations:** Department of Anesthesiology, Nihon University School of Medicine, 30-1 Oyaguchi Kamimachi, Itabashi-ku, Tokyo 173-8610, Japan

## Abstract

We report a patient with myotonic dystrophy who showed prolonged rocuronium-induced neuromuscular blockade, although with a fast recovery with sugammadex. During general anesthesia with propofol and remifentanil, the times to spontaneous recovery of the first twitch (T1) of train of four to 10% of control values after an intubating dose of rocuronium 1 mg/kg and an additional dose of 0.2 mg/kg were 112 min and 62 min, respectively. Despite the high sensitivity to rocuronium, sugammadex 2 mg/kg administered at a T1 of 10% safely and effectively antagonized rocuronium-induced neuromuscular block in 90 s.

## 1. Introduction

Myotonic dystrophy (MD), an autosomal dominant disorder, is the commonest of all myotonic syndromes, with an incidence of approximately 1 in 8000. It is characterized by progressive muscle weakness of the face, neck, pharynx, and distal limbs, with difficulty initiating movements and delayed muscle relaxation [1]. Careful anesthetic management is required for MD patients due to the likelihood of various coexisting disorders, such as cardiac conduction abnormalities, hypotension, diabetes mellitus, dysphagia, and malignant hyperthermia [2]. Changes in the sensitivity of these patients to neuromuscular blocking agents also require special consideration. In particular, the potential requirement of prolonged ventilatory support due to hypersensitivity to non-depolarizing neuromuscular blockade [3, 4], cardiac arrest provoked by succinylcholine [5], and neostigmine-induced myotonia [6] should be considered in MD patients. We present a patient with MD whose neuromuscular function was successfully managed with rocuronium and sugammadex during general anesthesia.

## 2. Case Presentation

A 37-year-old female patient with MD, weighing 55 kg and 154 cm tall, was scheduled for open resection of an ovarian tumor under general anesthesia combined with epidural anesthesia. Beside MD, her surgical history included retroperitoneal tumor resection under general anesthesia, although details about the surgery and the patient's perioperative condition were not known. Preoperative manual muscle tests revealed mild muscular weakness and myotonia in her upper limbs. She complained of mild difficulty in swallowing, although her respiratory efforts did not seem to be impaired. Moderate masseter muscle atrophy led us to predict difficulty with bag and mask ventilation during the induction of anesthesia. Routine preoperative blood tests were within normal ranges with no elevation of creatine kinase levels and no indication of liver or renal insufficiency. Arterial blood gas analysis at a F_I_O_2_ of 0.21 showed an arterial oxygen tension of 85 mmHg and carbon dioxide tension of 47 mmHg.

**Figure 1:**
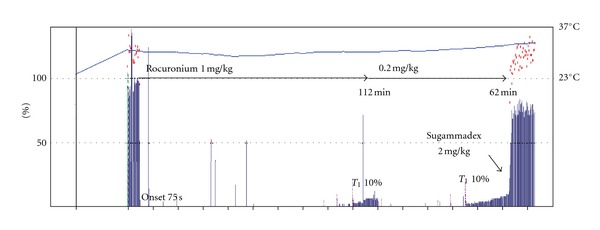
A serial recording of acceleromyography in a patient with myotonic dystrophy. Blue longitudinal bars show T1 height in the train-of-four responses, and red dots mean the train-of-four ratios. Marked prolongation in durations of rocuronium-induced neuromuscular block and rapid recovery from neuromuscular block after sugammadex administration are shown.

Premedication consisted of oral administration of 150 mg ranitidine the night before and on the morning of surgery. On arrival at the operating room, the patient was monitored with ECG, noninvasive blood pressure, and pulse oximetry. Epidural puncture and catheterization were performed at the Th12-L1 intervertebral space. General anesthesia was induced with fentanyl 2 *μ*g/kg and a target controlled infusion of propofol 4 *μ*g/mL (Terufusion TCI pump TE-371, Terumo, Tokyo Japan) while the patient received 100% oxygen through an anesthesia facemask. After loss of consciousness, the left ulnar nerve was stimulated at the wrist with supramaximal and square-wave stimuli of 0.2 ms duration, which was delivered in a train-of-four (TOF) mode at 2 Hz every 15 s. Contraction of the ipsilateral adductor pollicis muscle was measured using an acceleromyograph (TOF-Watch SX; Organon, Dublin, Ireland). Immediately after obtaining baseline levels of TOF responses, the patient received a bolus of rocuronium 1 mg/kg. Complete neuromuscular block was obtained 75 seconds after rocuronium administration, and the patient's trachea was intubated thereafter without any difficulty. Ventilation was controlled with a tidal volume of 500 mL and at a rate of 10/min. Anesthesia was maintained with propofol 2–4 *μ*g/mL, remifentanil 0.05–0.3 *μ*g/kg/min, and intermittent epidural injections of 0.375% ropivacaine. The first twitch (T1) of train of four recovered to 10% of control levels 112 min after administration of the intubating dose of rocuronium. At that time, rocuronium 0.2 mg/kg was administered to obtain complete neuromuscular blockade, as observed by absent TOF responses. The duration to spontaneous recovery to a T1 of 10% of control levels was also prolonged to 62 min (Figure 1). At the time of uneventful completion of the surgery, the rocuronium-induced moderate neuromuscular block was still present, and the observed TOF count was only 2. Sugammadex 2 mg/kg rapidly antagonized the neuromuscular block, such that the TOF ratio reached 0.9 in 90 s. Several minutes after discontinuation of propofol and remifentanil, the patient could breathe adequately and was extubated. Oxygen saturation measured by pulse oximetry remained at 100% while the patient received 100% oxygen via a facemask. Postanesthetic shivering that could have precipitated the myotonia was avoided by ensuring adequate intraoperative warming and temperature maintenance. Adequate postoperative analgesia was provided by continuous epidural injection of 0.2% ropivacaine without the addition of opioids. The postoperative course was also uneventful, and no respiratory complications were observed.

## 3. Discussion

Our patient exhibited a higher sensitivity to rocuronium-induced neuromuscular blockade. The time from administration of rocuronium 1 mg/kg until T1 spontaneously reached 10% of the control value was markedly longer in our patient as compared to patients with normal neuromuscular function (70 min [7]). The time taken for T1 to reach 10% in our patient (112 min) was measured during intravenous anesthesia using propofol and remifentanil, while the previous data was observed during anesthesia with sevoflurane [7], which is known to significantly prolong the duration of action of rocuronium to 1.5–2 times [8, 9]. Assuming that the values observed in the other study were potentiated by sevoflurane, the time from administration of rocuronium 1 mg/kg to the recovery of T1 to 10% of the control level observed in our MD patient seems to have been roughly doubled.

The response of MD patients to non-depolarizing neuromuscular blocking agents is controversial. Increased sensitivity [3, 4], normal response [4], and even resistance [10] to non-depolarizing neuromuscular block have all been reported. It is likely that the degree of severity of the pathology may determine the sensitivity to neuromuscular blockade [11]. To eliminate the risk of prolonged neuromuscular block and avoid the need for mechanical ventilation in the postoperative period in these patients, avoidance of the use or reduction in the dose of neuromuscular blocking agents is recommended [4]. However, vocal cord injury is a serious concern when tracheal intubation is performed without neuromuscular blocking agents [12]. In addition, inadvertent patient movement can be triggered if neuromuscular blockade during surgery is inadequate. More importantly, our patient had dysphagia associated with dysfunction of the pharyngeal muscles and the risk of regurgitation of gastric contents [11]. Furthermore, difficulty with bag and mask ventilation during induction of anesthesia was predicted because of the masseter muscle atrophy. Therefore, rapid sequence intubation using a high dose of rocuronium was planned to avoid aspiration pneumonia and difficult ventilation, despite the risk of prolonged neuromuscular blockade. Use of cisatracurium also seemed like a logical choice because the benzylisoquinoline compound constantly undergoes pH- and temperature-dependent Hofmann elimination in plasma and tissues [13]. Although the use of neuromuscular blocking agents without reversal has been shown to be a significant risk factor for postoperative respiratory complications [14], anticholinesterases should also be avoided in these patients so as to avoid evoking myotonia, even at the potential cost of residual neuromuscular blockade postoperatively. These contraindications to the use of non-depolarizing muscle relaxants and their reversal agents and the availability of sugammadex, which can promptly antagonize rocuronium-induced neuromuscular block even in myasthenic patients [15], partly contributed to our decision to use high-dose rocuronium. In fact, reversibility of rocuronium-induced neuromuscular block with sugammadex has been proved to be adequate even in MD patients with a high sensitivity to rocuronium. In such cases, however, the dosing of rocuronium and sugammadex should be individually optimized by neuromuscular monitoring because recurarization may occur after administration of a lower dose of sugammadex [16].

Acceleromyography was very useful to evaluate the onset of and recovery from rocuronium-induced neuromuscular block in our MD patient, although it may underestimate the degree of neuromuscular block during recovery on the negative side [11]. It has been reported that T1 is still recovering from neuromuscular block even when the TOF ratio reaches 0.9 after reversal with sugammadex [17]. Therefore, when residual neuromuscular block is suspected by clinical signs of respiratory insufficiency and inadequate muscular strength, additional doses of sugammadex should be considered.

It is likely that not only rapid reversal of rocuronium-induced neuromuscular block with sugammadex but also short-acting intravenous anesthesia with propofol and remifentanil and postoperative analgesia without opioids all contributed to the rapid recovery of respiratory function seen in our patient. Given the importance of the marked susceptibility of MD patients to anesthetics, which may cause apnea and respiratory depression [18], careful titration of propofol and remifentanil by a target-controlled infusion is thought to be appropriate when anaesthetizing MD patients.

In conclusion, the combination of rocuronium and sugammadex may allow safe and effective management of neuromuscular function during general anesthesia in patients with MD. Further systematic studies are warranted to verify the safety and efficacy of perioperative use of rocuronium and sugammadex in MD patients.
